# Coming out: the experience of LGBT+ people in STEM

**DOI:** 10.1186/s13059-017-1198-y

**Published:** 2017-04-04

**Authors:** Ben Barres, Beth Montague-Hellen, Jeremy Yoder

**Affiliations:** 1 Department of Neurobiology, Stanford University School of Medicine, Fairchild Building D235, 299 Campus Drive MC5125, Stanford, CA 94305 USA; 2 Research Services Unit, The University Library, University of Sheffield, Sheffield, S10 2TN UK; 3 Department of Forest and Conservation Sciences, University of British Columbia, Vancouver, BC V6T 1Z4 Canada

## Abstract

Continuing with our Q&A series discussing issues of diversity in STEM fields, *Genome Biology* spoke with three openly LGBT+ researchers on their experiences in biology.

Despite growing acceptance of lesbian, gay, bisexual, transgender people (LGBT; subsequently LGBT+ to encompass a spectrum of genders and sexualities), discrimination and alienation remain. A recent report [1] has shown that many STEM workplaces and professional culture can exclude people who identify as LGBT+ (Fig. 1). *Genome Biology* spoke to Beth Montague-Hellen, Jeremy Yoder, and Ben Barres about their personal experiences.

**Fig. 1:**
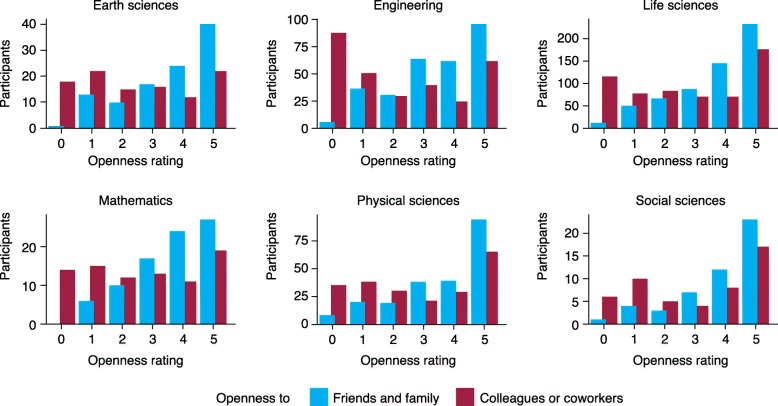
LGBT+ scientists aren’t all that out of the lab closet. STEM professionals rate their openness about LGBT+ identities in different contexts, from 0 (no one knows) to 5 (everyone knows) [1]

## You’ve each spoken openly and publicly about your experiences. What would you say influenced your choice about coming out in the workplace/as a graduate student?

BB: About 2 years after I started my lab at Stanford, I realized that I was transgender and decided to transition from female to male. As this was 23 years ago, a time when there had been few openly transgender scientists, I did initially worry about potential repercussions to my career. However, after discussing with several close colleagues, I decided that my friends and colleagues would be supportive. Indeed from the time I transitioned, all of my friends, students, and colleagues have been very strongly supportive. I have been far happier living openly in the gender identity that corresponds with my internal feelings and have never regretted this decision.

BMH: I’ve never really been in a position to hide my sexuality; for most of my adult life it’s mostly been a case of confirming that I was gay rather than “coming out.” As a PhD student I was the LGBT SU officer at Sussex University, so I had a large profile as an LGBT+ student, I knew other LGBT+ PhD students and the campus was very welcoming anyway. In other institutions I have not always felt that it was as easy to come out and talk about my life as an LGBT+ person, but I’ve also always felt that it was important that I did, to provide a visible person other LGBT+ students and researchers could identify.

JY: Well, for me, graduate school was the stage in my life when I first met openly gay people and made gay friends. It’s embarrassing in retrospect, but that’s pretty much all there is to it. I’d had a conservative upbringing and hadn’t really been able to picture a future in which I could be fully myself and have the life I wanted—and then I met well-adjusted, happily out folks and learned about senior biologists who’d been out for decades. It’s no exaggeration to say that science was my “safe space.”

## Can you comment on any barriers or unconscious biases you have experienced as a scientist that are not experienced by straight/cis-gendered people?

BB: Having lived life first as a woman and then as a man, like most transgender people, I am very aware of the different ways that society often treats people simply based on their gender. These experiences have made me very aware that women scientists still face substantial barriers in their careers that men do not generally face. These barriers indeed sometimes come from unconscious bias, but also sometimes from quite conscious bias. I have heard many senior male scientists say they do not like to take women trainees for fear they may have a baby while in their lab. The barriers also come from the persisting academic structures that were initially set up for men by men. The tenure clock is a very good example of such a barrier as it strikes just as women are in their final reproductive years. We should not eliminate tenure, but we should find a way to let all faculty make this tenure at an appropriate rate. A solution I favor is to grant tenure on the day a faculty member begins their assistant professorship. They would still have to make promotions but they could do this at the rate that was right for their circumstances. Yes the downside would be that occasionally people would be tenured inappropriately but in a supportive environment this is unlikely to happen often as young scientists who win job searches have typically been highly successful as PhD students, again as postdocs, and then win a highly competitive search—how many times does a young scientist have to prove herself? Granting tenure on the day assistant professorship starts would also have an enormous upside for men as well as women, as all young faculty would not be put in a risk-averse mode at the very start of their careers.

BMH: Regarding unconscious biases, I’ve felt that my butch/masculine identity has actually helped me in my career. Bioinformatics tends to be one of the more male-heavy disciplines in the biological sciences and I’ve often felt that it may be easier for me than for someone who was more feminine. However, it hasn’t always been rosy. I’ve found that while political debates regarding LGBT+ rights are happening colleagues often feel free to discuss these issues as if you are not there or as if these issues don’t have a direct bearing on your life. This was particularly the case for me while a postdoc during the UK equal marriage debates, so much so that at times I would actively try to avoid collaborating with or interacting with colleagues who I knew might have views opposed to the progression of LGBT+ rights.

JY: I honestly haven’t been aware of bias or career barriers resulting from my identity as a gay man. I want to emphasize, though, that I’ve been fortunate to be in a field that tends to attract liberal folks—evolutionary biology. I also have advantages stemming from other elements of my identity that make life easier—I’m white, my gender identity corresponds with what I was assigned at birth, and I had a middle-class upbringing by parents who both went to college. In part because I knew my experience might be unrepresentative, I went looking for data on LGBTQ representation and experiences in science a few years ago, and I stumbled into a wonderful collaboration with a friend who is a social scientist, Allison Mattheis. We surveyed STEM professionals who identify as lesbian, gay, bi, trans, or otherwise queer, and called it the Queer in STEM project [2]. We found that experiences can vary quite a bit in different STEM fields and in different workplaces [1]. Of the more than 1400 people who participated, a large majority told us they were open about their LGBTQ identity in their personal lives; but a substantial fraction also said that none of their colleagues, coworkers, or students knew. That’s bad for psychological well-being, career satisfaction, and productivity.

## What have been your biggest challenges and greatest opportunities in your career?

BB: The greatest challenge in my career was deciding to work on a cell type—glia—that was widely considered to be unimportant and therefore largely neglected. Given that glia constitute most of the cells in the human brain, I was just curious to know more about their function. It seemed obvious to me that they were important, but my grant reviewers often disagreed! Selecting to work on glia was also a substantial challenge as it meant that I had to spend a lot of effort developing tools to study them. After 35 years of working on glial cells, the times have changed and due to our work and many others it is now clear that neuron–glial interactions are critically important in both health and disease. So in a way deciding to work on glia turned out to be both the biggest challenge but also the greatest opportunity in my career.

BMH: At the end of my first postdoctoral job I decided to move to America to work at Rutgers University for a year. Given the current political climate I’m glad I went then rather than now, but the decision to move to a country where I felt many people were less welcoming of my sexuality and gender expression was a rather scary one. Due to laws regarding the definition of a spouse, my wife stayed in England, which made the decision even more difficult; however, I believe that taking that jump and facing my fears was an amazing experience, greatly helped my career and, if anything, made my relationship stronger.

JY: I think my biggest challenges have been the ones shared by most scientists of my generation—building a research reputation in an era of tight scientific funding and an ever-more-competitive academic job market. I just accepted a faculty position that I’m very excited about, but it took more than 6 years of postdoc work, two cross-country moves, and scores of applications.

## What more can be done to encourage and support LGBTQIA scientists?

BB: As an openly transgender scientist for the past 23 years, I have come to realize that having openly LGBT role models intensely matters to the young generation. I have been contacted by many young scientists struggling with whether to be open about their sexual orientation or gender identity. Many have commented that it has really helped them to decide to be open to know of other LGBT scientists who are open. I am always surprised that even in the Bay Area there are many LGBT students and scientists that are still in the closet. I do not fault these people as their experiences may have been different than mine, they may live in different parts of the country where bigotry may be more prevalent, and they may have experienced more barriers in their lives. But I would argue that presently the advantages far outweigh the risk. The vast majority of academics are highly supportive. It is very difficult to live life in a closet. It does not make sense to do this because of an occasional bigot, and of course as more LGBT people are open, ignorance is lessened and the path forward gets easier for all. I always counsel young scientists to be open about who they are and I have yet to have anyone tell me they regretted this decision.

BMH: While I think institutions have a duty to ensure that their policies protect and encourage LGBT+ scientists, I believe that one of the best things that can be done is for us to stand up as a community to say “Here we are.” I’ve instigated several projects to this effect. The first project is the LGBT STEM blog [3] and Twitter account (@LGBTSTEM)—a collection of profiles of LGBT+ scientists which is growing all the time. The second is the LGBTSTEMinar [4], a conference which has been held at the University of Sheffield for the last 2 years showcasing science, technology, engineering, and maths research being carried out by LGBT+ individuals and encouraging networking within the community.

JY: A key finding from the Queer in STEM survey project is that concrete employer policies really do seem to make a difference—participants who said their employers provided same-sex partner benefits or support for name changes associated with gender transitions, for instance, were more likely to be out of the closet at work. We also found an interesting pattern that participants were more open if they worked in STEM fields with better representation of women. That suggests to me that diversity could have a multiplicative effect, and as the “typical” image of a scientist comes to encompass more types of people, STEM will become more welcoming to an even broader array of identities. There’s a lot of work to do, but I’m optimistic to see the scientific method can be used to help make scientific careers accessible to more people—which will ultimately make science work better for everyone.

## Box 1 Contributors’ research interests

**Ben Barres (BB):** My lab is focused on understanding the nature of neuron–glia interactions in health and disease. Recent studies have focused on understanding the role of synaptic phagocytosis by astrocytes in CNS synaptic plasticity underlying learning and memory, how oligodendrocytes differentiate and myelinate, the roles of microglia, and reactive astrocytes. We have recently discovered that reactive astrocytes are highly neurotoxic after CNS injury and in neurodegenerative diseases; we are purifying and identifying this neurotoxin to further investigate its contribution to neuronal death and synapse loss in these diseases.

**Beth Montague-Hellen (BMH):** My research interests have mostly been in the area of mammalian comparative genomics, particularly regarding non-coding sequences such as transposable elements and regulatory elements. However, recently my interests have started moving towards research data management and developing ways to help and encourage other researchers to share data.

**Jeremy Yoder (JY):** I study the ways in which interacting species shape each other’s evolution, and how adaptation to different living communities and habitats creates and maintains biodiversity. Most of my research has focused on natural populations of plants—I’ve studied the intimate pollination mutualism of Joshua trees and adaptation to climate and nitrogen-fixing symbiotic bacteria by a Mediterranean wildflower called barrel medick, and I’m currently working on a study of the genomic basis of adaptation to climate in lodgepole pine and interior spruce
